# A novel plate design versus two conventional miniplates for treatment of mandibular angle fractures (a pilot randomized controlled clinical trial)

**DOI:** 10.1186/s12903-026-08474-5

**Published:** 2026-05-09

**Authors:** Peter N. El-Masry, Nevein S. Mohamed, Mohamed E. Saber

**Affiliations:** Oral and Maxillofacial Surgery Department, Faculty of Dentistry, Alexandria University, Champollion St., Azarita, Alexandria, 21527 Egypt

**Keywords:** Mandibular angle fracture, Miniplate, Sagittal split plate, Bone density, Osteosynthesis

## Abstract

**Background:**

Among facial skeletal injuries, mandibular fractures are highly prevalent, second after nasal bone fractures. Consequently, a lot of research has focused on treatment modalities for these fractures, to ensure faster healing & rigid fixation. One of these modalities is the application of the Sagittal Split plate at the mandibular angle. The objective of this study is to compare the clinical and radiographic outcomes of the use of a novel Sagittal Split Osteotomy Plate (SSOP) design versus conventional two-miniplate fixation in the treatment of mandibular angle fractures, and to assess the biomechanical stability of the new design.

**Materials and methods:**

In this pilot randomized controlled trial, 16 patients with mandibular angle fractures were randomly assigned (1:1 allocation ratio) using computer-generated block randomization into two groups. Group A (8) patients were treated using the new plate modified from Sagittal Split plate, and Group B (8) patients were treated using two conventional miniplates. Mechanical test bench was performed to measure plate strength & mechanical stability of the new plate design. Clinical evaluations were performed at 24 h, one, four, six and twelve weeks postoperative to assess operating time, pain, occlusion, wound healing & interincisal mouth opening. In addition, radiographic assessments were conducted immediately postoperatively and at three months to evaluate the mean bone density at the fracture site.

**Result:**

The study group showed reduced operating time. No statistically significant differences between the groups regarding occlusion, wound healing, postoperative pain and interincisal mouth opening. A statistically significant difference was observed regarding mean bone density of the study group after 3 months. The mean displacement of the mechanical test was 1.46 mm under 60 N of the new plate.

**Conclusion:**

Within the limitations of this pilot study, the novel SSOP demonstrated reduced operative time and comparable short-term clinical outcomes to conventional double-miniplate fixation. Radiographically, higher bone density was observed at three months, and the prototype demonstrated acceptable baseline biomechanical outcomes during pre-clinical testing. While the SSOP represents a potential fixation alternative, larger longitudinal studies remain necessary to evaluate long-term clinical outcomes and to directly compare this prototype biomechanically against other established plates used for mandibular angle fractures.

**Trial registration:**

This clinical trial was prospectively submitted for registration to ClinicalTrials.gov prior to the enrollment of the first participant (identifier NCT07103590; official public posting August 5, 2025).

**Supplementary Information:**

The online version contains supplementary material available at 10.1186/s12903-026-08474-5.

## Background

Mandibular fractures rank second only to nasal bone fractures in the frequency of facial bone injuries [1, 2]. Despite being the strongest bone of the facial skeleton, the mandible’s prominence and mobility make it more vulnerable to trauma from road traffic accidents, violence, and falls [3]. Mandibular angle fractures (MAFs) account for approximately 30% of facial fractures [4]. This region consists of structurally weak areas due to the presence of the third molar and the abrupt change in the bone’s cross-sectional area [5]. If left untreated, injuries can lead to significant functional impairments affecting mastication, deglutition, and speech [6].

The management of mandibular fractures aims to regain pre-injury strength, prevent infection, and restore function with minimal patient discomfort [7]. Treatment modalities have evolved from closed techniques, such as Intermaxillary Fixation (IMF), to rigid internal fixation (RIF), which was introduced in the late 1970s [8]. Modern open reduction methods using miniplates allow for rapid functional recovery and significantly shorter healing periods [9]. However, despite these advancements, there is currently no consensus regarding the optimal fixation scheme for angle fractures [10, 11].

Miniplate fixation based on Champy’s principles has gained popularity due to its ease of manipulation and reduced surgical trauma [12–14]. Nonetheless, conventional miniplates may lack the rigidity required to withstand torsional movements under functional load, potentially leading to complications such as non-union or infection [15]. Consequently, restricted jaw function or a short period of postoperative IMF is often recommended to compensate for this reduced stability [3].

In 2014, Suer et al. [16] introduced a modified miniplate. Although this design improved resistance to lateral displacement, it lacks the mechanical capacity to withstand complex tension and compression forces when anchored at the inferior border. Furthermore, while standard Sagittal Split Osteotomy Plates (SSOP) are highly effective in elective orthognathic surgery, their traditional linear configuration is not optimized for trauma cases requiring multiplanar tension neutralization [17]. Similarly, although 3D miniplates offer enhanced stability, they often present challenges when adapting to comminuted or oblique fractures in addition to high cost. To address these limitations, a novel design based on the Sagittal Split Osteotomy Plate (SSOP) was developed. Anchored in the dense basal bone, its vertical extensions reach into the superior tension zone to create a multiplanar geometric frame. This hybrid design bridges the simplicity of conventional miniplates with the rigidity of 3D systems, distributing functional loads to effectively neutralize both tensile and torsional forces.

The primary objective prioritized the assessment of fracture stability and radiographic bone density. Secondary objectives focused on functional recovery, including postoperative pain, the occlusion stability, maximal interincisal opening (MIO), and the incidence of wound healing complications, as well as surgical efficiency based on mean operative time. Furthermore, the study aimed to validate the mechanical integrity of the novel design through two modalities: pre-clinical in vitro testing under simulated functional loads, and clinical 3D superimposition of immediate and 3-month postoperative scans to quantify in vivo hardware displacement. The null hypothesis posits that there is no statistically significant difference in radiographic bone density or clinical outcomes between the two groups. The alternative hypothesis assumes that a statistically significant difference exists.

## Methods

### Study design and ethical considerations

This prospective, randomized controlled clinical trial (1:1 allocation) was conducted at the Department of Oral and Maxillofacial Surgery, Alexandria University, in strict accordance with CONSORT 2025 guidelines. Clinical enrollment commenced on July 17, 2025, following the prospective submission on ClinicalTrials.gov (NCT07103590; official public posting August 5, 2025). A block randomization was performed with block size of four using computer-generated random numbers to ensure unbiased group assignment.

Written informed consent was obtained from all participants following a transparent discussion of the protocol and alternative treatments including intraoral open reduction and closed reduction techniques. Patients were explicitly counseled on the risk-benefit profile of the selected extraoral approach, specifically weighing the inherent risks of scarring and marginal mandibular nerve injury against the biomechanical necessity for optimal visualization and rigid fixation at the inferior border.

### Participants

Participants were recruited from the Outpatient Clinic of the Alexandria University Teaching Hospital.

### Inclusion criteria [18]

Adult patients (20–50 years) presented with disturbed occlusion requiring open reduction for severely displaced, unfavorable, and non-comminuted mandibular angle fractures. Acknowledging the rarity of strictly isolated angle fractures, patients with associated contralateral fractures (e.g., Parasymphyseal or body) were included, provided the contralateral site was treated with standard internal fixation to prevent biomechanical interference with the study variable.

### Exclusion criteria

Patients with medical conditions that contraindicate general anesthesia, those with pathological or non-recent fractures, non-displaced fractures or active infection at the fracture site.

### Study variables and outcome assessment

The primary predictor variable was the method of functionally stable internal fixation (novel SSOP versus conventional two-miniplate). Primary outcome variables evaluated mechanical success, specifically clinical fracture stability was assessed via bimanual palpation immediately post-operatively and at follow-ups. Stability was strictly defined as the complete absence of perceptible pathological mobility across the fracture site and radiological bone healing, quantified in Hounsfield Units (HU) via computed tomography (CT) immediately post-operation and at three months.

Secondary outcomes assessed reduction accuracy (radiological alignment), functional recovery, and surgical efficiency. Radiological alignment was quantified on postoperative CT scans by measuring residual step deformities in both the vertical (coronal plane) and horizontal (axial plane) dimensions. Clinical efficacy was evaluated through the stability of postoperative occlusion, maximal interincisal opening (MIO), pain assessment, and the incidence of complications (e.g., infection, dehiscence). Surgical efficiency was defined by the total operative time. Finally, to validate the structural integrity of the novel prototype, in vivo hardware displacement was quantified exclusively in the study group via 3D superimposition of the immediate and 3-month CT scans.

### Sample size estimation

The sample size was calculated based on the primary outcome of radiographic bone density (HU). This quantitative, continuous metric was selected because it directly correlates with the mechanical stability of the fixation construct, offering adequate statistical power without requiring the unrealistically large cohorts needed to detect rare clinical complications. Based on previous literature [19], the expected percent change in bone density after three months was 584.89 ± 74.17% in the two-miniplate group and 777.32 ± 124.79% in the modified sagittal split plate group. Assuming a 95% confidence level and 80% study power and utilizing the highest standard deviation (124.79) for a comparison between independent means, the minimum sample size was calculated to be 8 cases per group (effect size = 1.542). Thus, the total required sample size was 16 cases.

### Allocation concealment

Allocation concealment was ensured using sequentially numbered, opaque, sealed envelopes (SNOSE). These were maintained by independent personnel and opened only immediately prior to surgery to strictly conceal the intervention assignment from the operator.

### Blinding

Due to the surgical nature, blinding the operator was not feasible. However, a single-blind protocol was maintained to minimize bias: participants were blinded to their group allocation, as both groups underwent an identical extraoral approach and postoperative regimen. Furthermore, clinical parameters (pain, mouth opening, and infection) were evaluated by an independent, blinded assessor, who was unaware of the group assignment, ensuring objective data collection, with inter-observer reliability verified to ensure data consistency.

### Study design: randomized controlled clinical trial (Fig. 1)

**Fig. 1:**
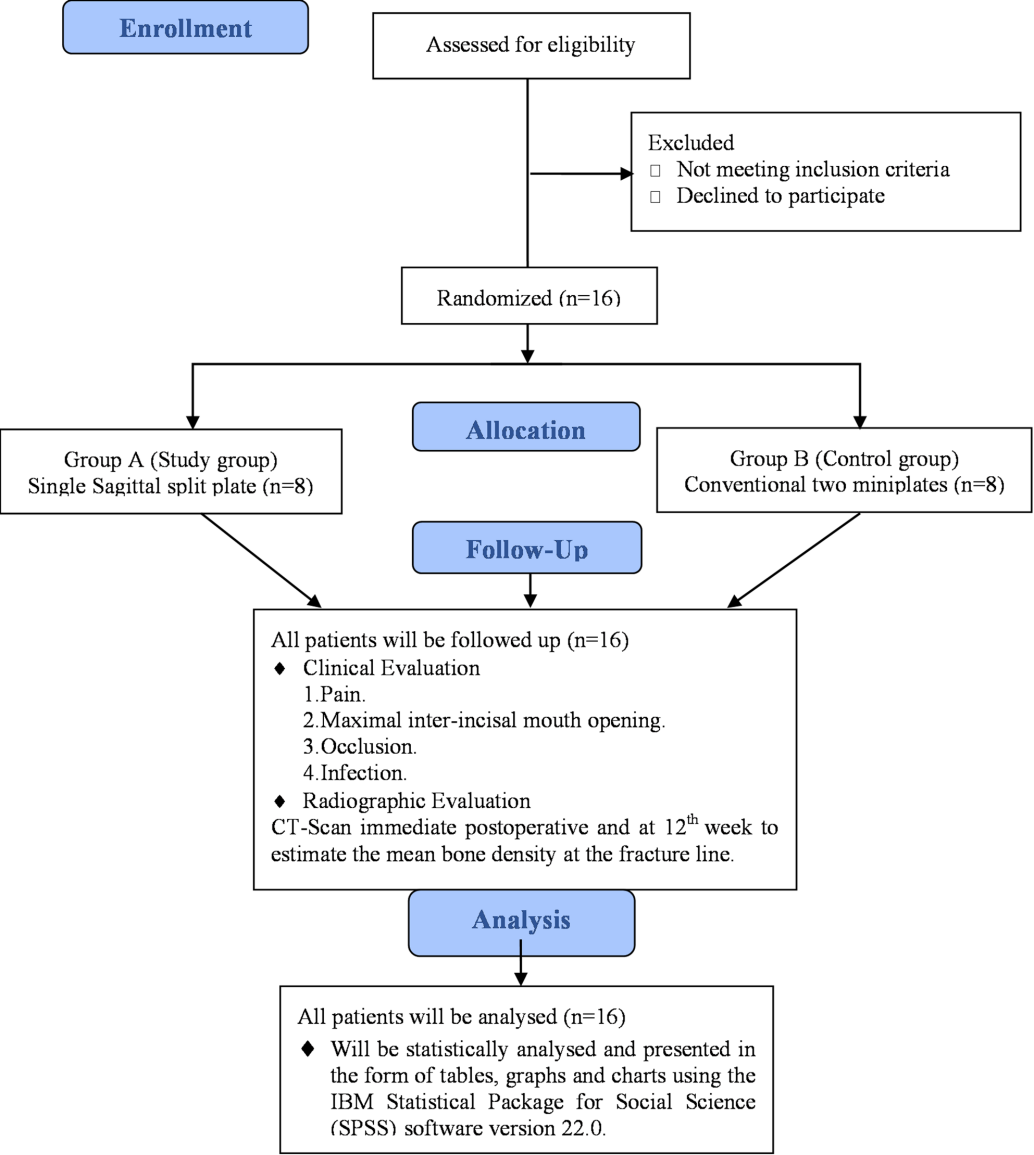
A flowchart summarizing the study design

#### Intervention: novel plate design

**Fig. 2:**
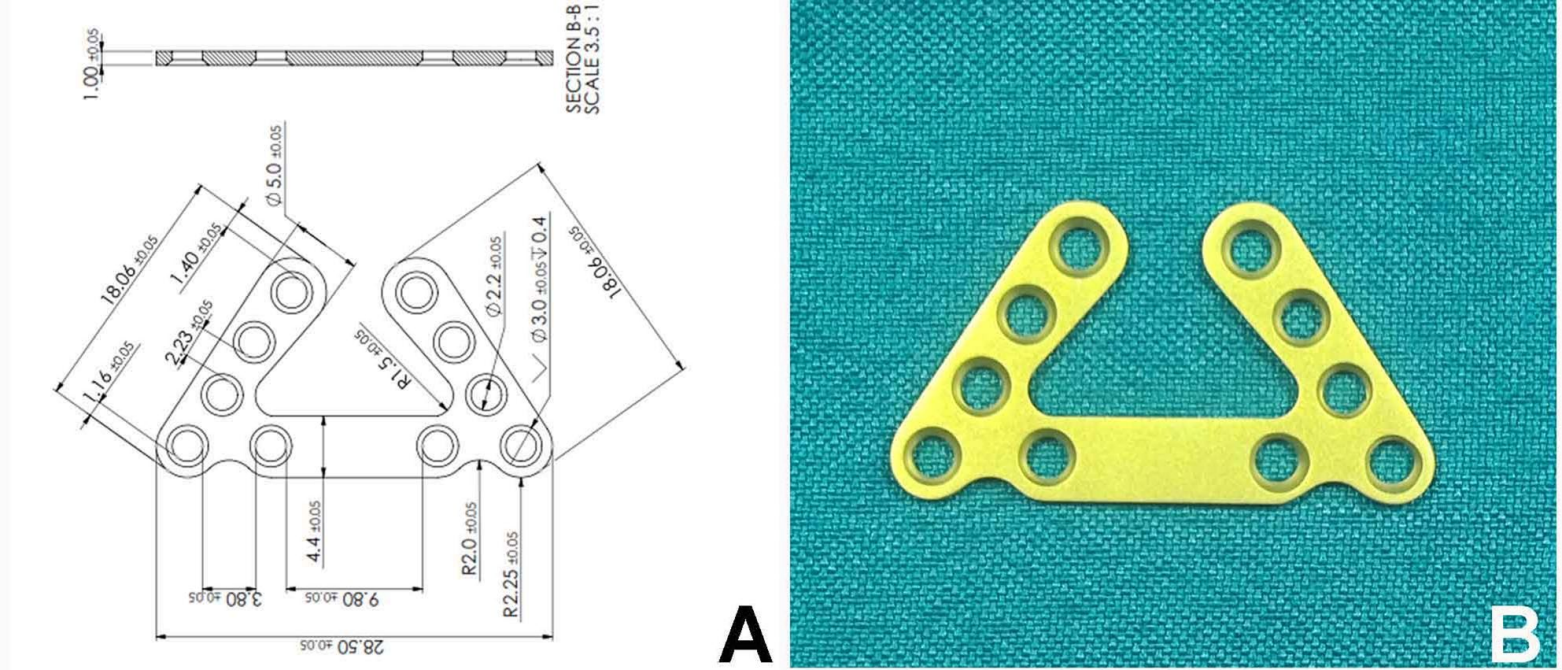
**A** New design of sagittal split osteotomy plate. **B** Printed design

The standardized SSOP prototypes were fabricated from commercially pure Grade 4 Titanium (complying with ASTM F67 standards for surgical implant applications) with a thickness of 1.0 mm. The plate features a trapezoidal geometry (18 mm length, 28 mm width) with a 60-degree internal angle and ten fixation holes: four in the base and three in each arm. The prototypes were produced via precision CNC milling at the Department of Production Engineering, Faculty of Engineering, Alexandria University, to maintain strict dimensional tolerance. It is secured at the inferior border (compression zone) with arms extending into the tension zone, bridging the fracture line with 2.0 mm bicortical screws (8 mm length) to resist multi-planar forces (Fig. 2A, B). Following manufacturing, the plates underwent ultrasonic cleaning and were sterilized via autoclaving at 121 °C for 20 min prior to clinical use. The manufacturing and clinical application of these investigational prototypes strictly adhered to the regulatory compliance guidelines established by the Egyptian Ministry of Health and the Research Ethics Committee of Alexandria University.

#### Pre-clinical biomechanical test

**Fig. 3:**
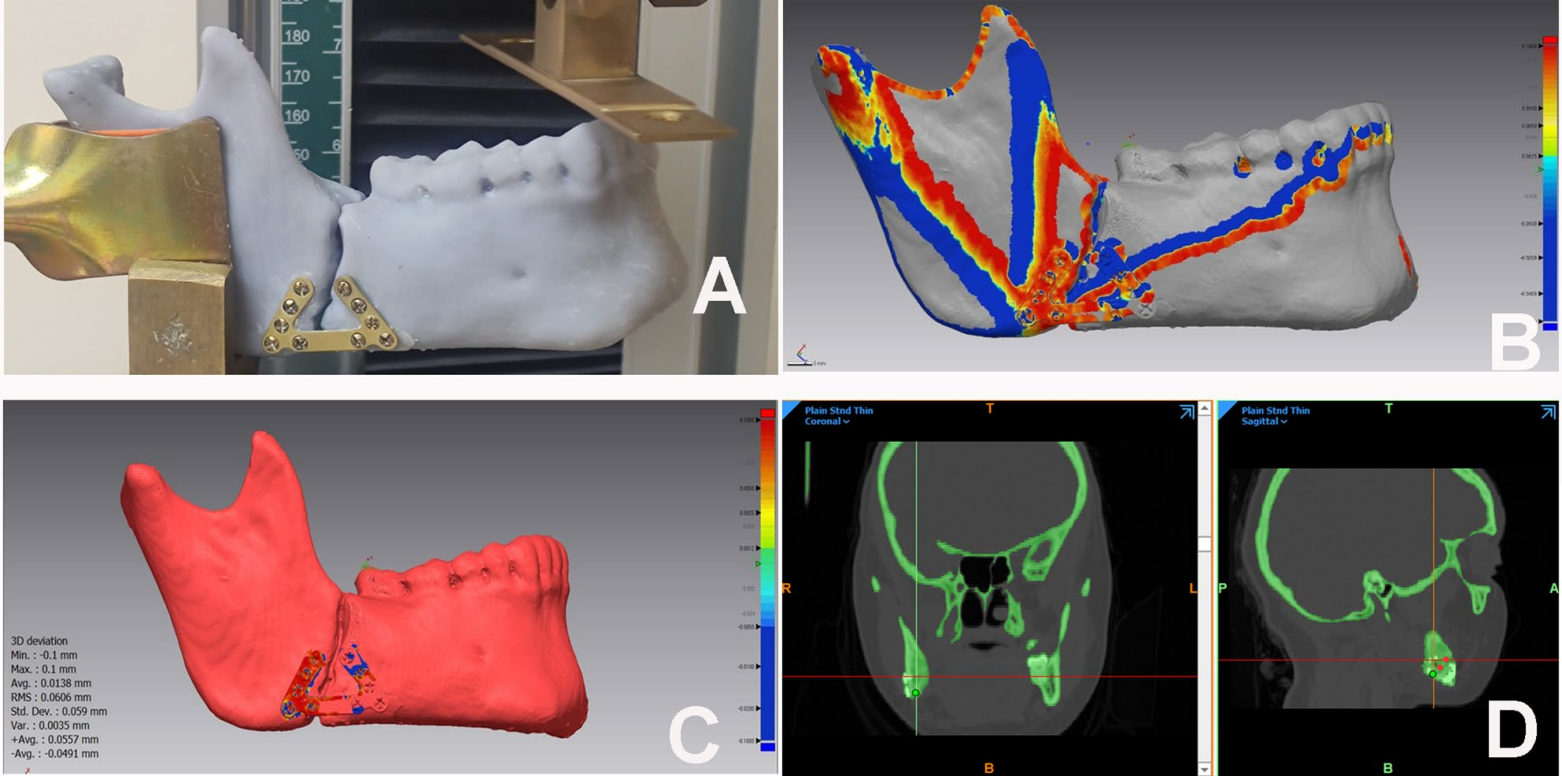
**A** Hemimandible replica under Mechanical test **B** shows the root mean square (RMS) of the new plate, which was used to determine the extent of plate deviation before and after the mechanical test. This RMS value was 60 microns **C** displays the hemimandible replica before and after the mechanical test, the color mapping indicates deviation from the reference data: blue areas show the measured data lying underneath the reference surface, while yellow to red areas indicate the measured data is in front of the reference surface, highlighting the most deviated parts. **D** ROI Diagram, Standardized placement of three fixed Point-ROIs (superior, middle, and inferior thirds) along the fracture line in Coronal & Sagittal Cuts postoperative and their average was calculated to determine the mean bone density using MIMICS software

Prior to clinical application, Polymeric Mandible Mechanical Testing (PMMT) was conducted to validate the structural integrity of the new design. DICOM datasets from five mandibular angle fracture cases were processed in MIMICS software to generate STL files, which were subsequently 3D-printed (Epax 3D 4K 6.6) using variable-density ebax resin to simulate cortical and trabecular bone structure. The fractured hemimandible replicas were reduced and fixated with the novel SSOP design at the inferior border. Mechanical testing was performed using a universal testing machine (5ST, Tinius Olsen, England). To ensure reliability and eliminate measurement bias, three iterations were conducted for each replica. Samples were secured in custom 3D-printed holders and subjected to progressive uniaxial loading up to 60 N applied to the incisal region (Fig. 3A). Displacement was quantified both mechanically and digitally; pre- and post-loading states were scanned (T710, Medit, South Korea) and superimposed using Geomagic Control X software to calculate 3D deviation.

### Presurgical phase

#### Preoperative assessment

A comprehensive preoperative evaluation was conducted for all participants, beginning with the collection of demographic data and a detailed history of the trauma, including etiology, mechanism, and timing of injury [20]. A systematic clinical examination was performed to assess soft tissue integrity, neurosensory status of the mental nerve, and occlusal disturbances [21]. Specific attention was given to identifying bony crepitus, step deformities, and functional limitations [18, 21]. To confirm the diagnosis and plan the surgical approach, radiographic assessment was completed using Computed Tomography (CT) scans to analyze fracture line patterns, the degree of displacement, and tooth involvement [21]. Crucially, the inferior alveolar canal was mapped using coronal and sagittal cross-sections to determine its apico-coronal position and measure the available bucco-lingual bone thickness. This allowed for the precise planning of safe screw trajectories, directly guiding the intraoperative ‘skip-hole’ technique to prevent iatrogenic nerve injury.

### Surgical procedure [22]

#### Preoperative preparation

Prior to the surgical intervention, all participants have done all the necessary laboratory investigations to obtain anesthesiologic clearance. Patients were instructed to strictly adhere to a fasting protocol for a minimum of eight hours before surgery. Prophylactic intravenous antibiotics and appropriate analgesics were initiated 12 h preoperatively to minimize risk of postoperative infection.

#### Surgical protocol

Under general anesthesia, a standard submandibular approach was utilized. A 4 cm incision was placed 1.5–2 cm inferior to the mandibular angle to protect the marginal mandibular nerve. Dissection proceeded through the platysma and investing fascia, identifying and protecting the facial vessels and submandibular gland capsule. The pterygomasseteric sling was incised to expose the fracture. Following temporary maxillomandibular fixation (MMF) to restore occlusion, fractures were reduced [23].

#### Fixation

**Fig. 4:**
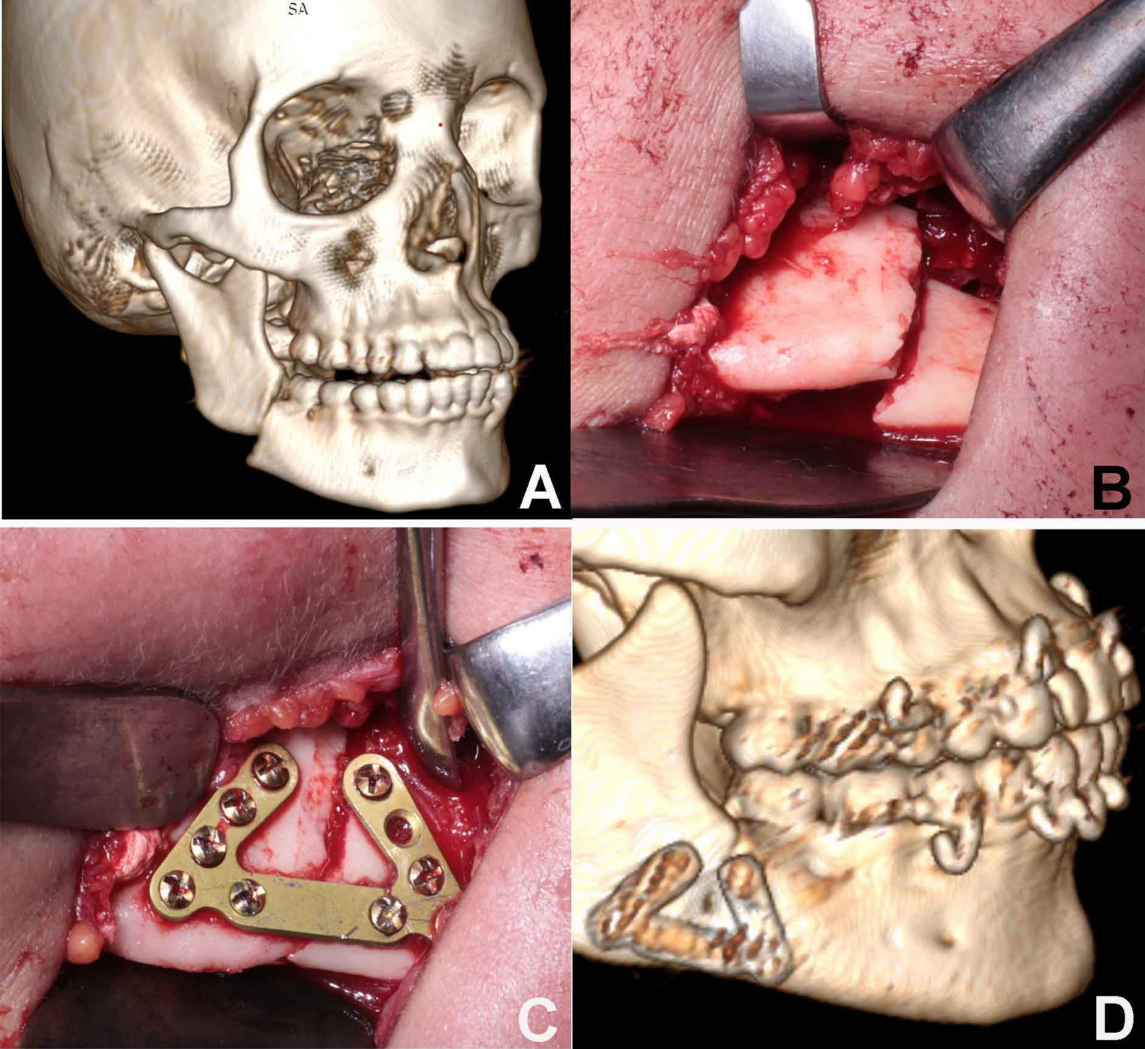
**A** Preoperative 3D. **B** Fractured segments before reduction. **C** New design plate in place after fracture reduction. **D** Postoperative 3D with the new plate

**Fig. 5:**
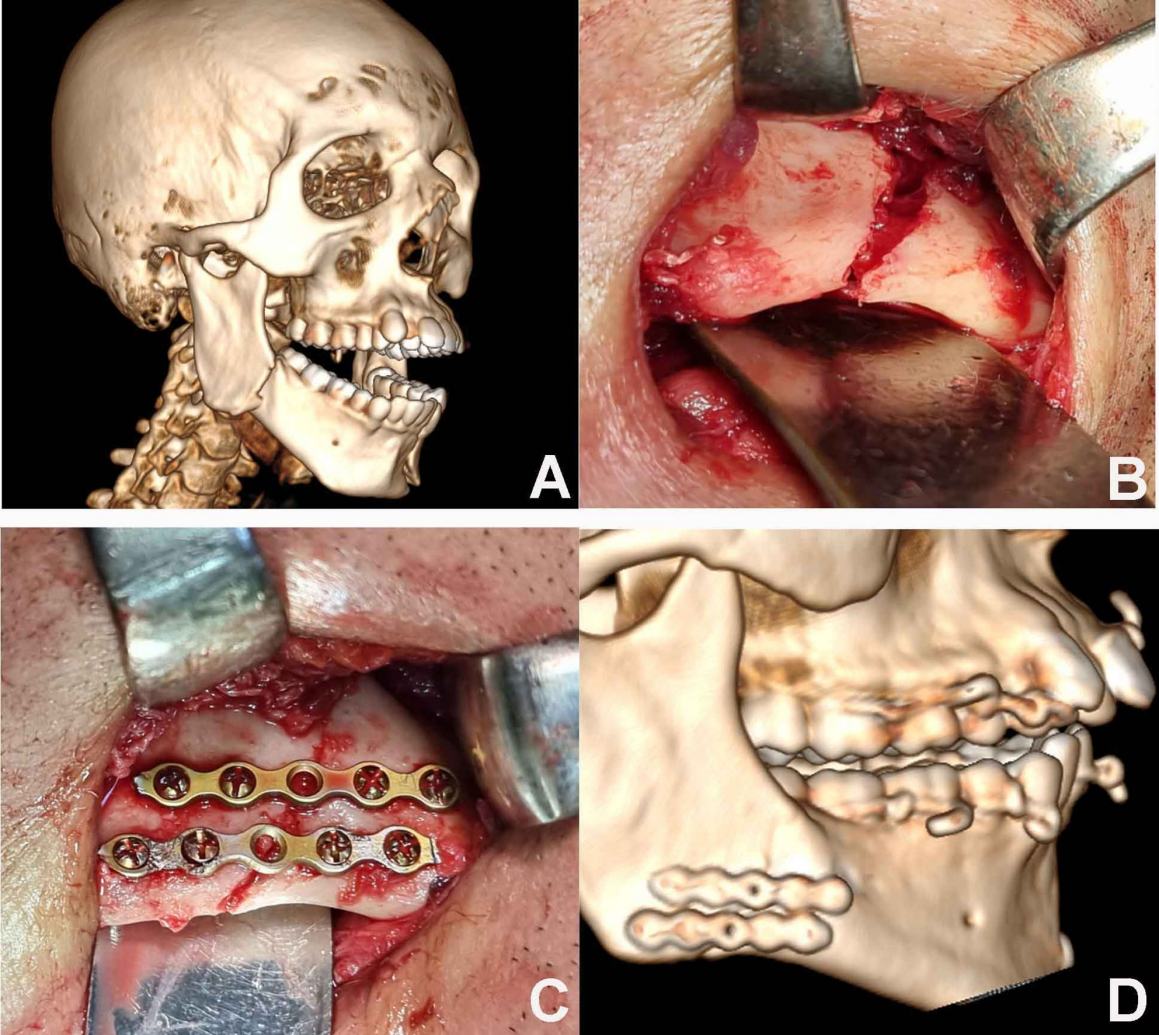
**A** Preoperative 3D. **B** Fractured segments before reduction. **C** Two conventional miniplates in place after fracture reduction. **D** Postoperative 3D with two miniplates

In the Study Group (Group A), the novel plate was adapted and secured at the inferior border with its arms extending superiorly (Fig. 4). In the Control Group (Group B), fixation was achieved using two conventional 2.0 mm miniplates placed at the inferior border (Fig. 5). MMF was released, and occlusion was verified before closing the wound in layers using 3- 0 Vicryl and 5- 0 Prolene sutures. Operative time from reduction to fixation was recorded.

For associated contralateral fractures, an anterior-to-posterior sequence was utilized. Following MMF, the contralateral fracture was fixed first using conventional miniplates to restore arch width, ensuring the angle fracture was evaluated within a stable arch.

#### Early postoperative care & postoperative medication

Immediately after surgery, all participants were instructed to apply extraoral ice packs for the first 12 h to minimize swelling. The postoperative pharmacological protocol included prophylactic intravenous Cefotaxime (1 g every 12 h) for the first 24 h, followed by a five-day course of oral Amoxicillin/Clavulanic acid (1 g twice daily) and Metronidazole (500 mg every 8 h). Patients also received alpha-chymotrypsin ampoules (once daily) and Diclofenac Potassium (50 mg every 8 h) for five days, to manage inflammation and pain. Additionally, all patients were advised to maintain strict oral hygiene using chlorhexidine mouthwash and to adhere to a soft, high-protein, high-calorie diet for four weeks postoperatively.

### Follow-up phase

Postoperative clinical follow-up evaluated pain using a standard 10-point Visual Analogue Scale (VAS) [24] and maximal interincisal opening (MIO) via a millimeter ruler [25]. Occlusal stability was verified by assessing the maximal intercuspal position for correct molar and canine relationships, noting any discrepancies such as anterior open bite [26]. Soft tissue healing was continuously monitored for signs of surgical site infection, wound dehiscence, or hardware exposure [27].

**Fig. 6:**
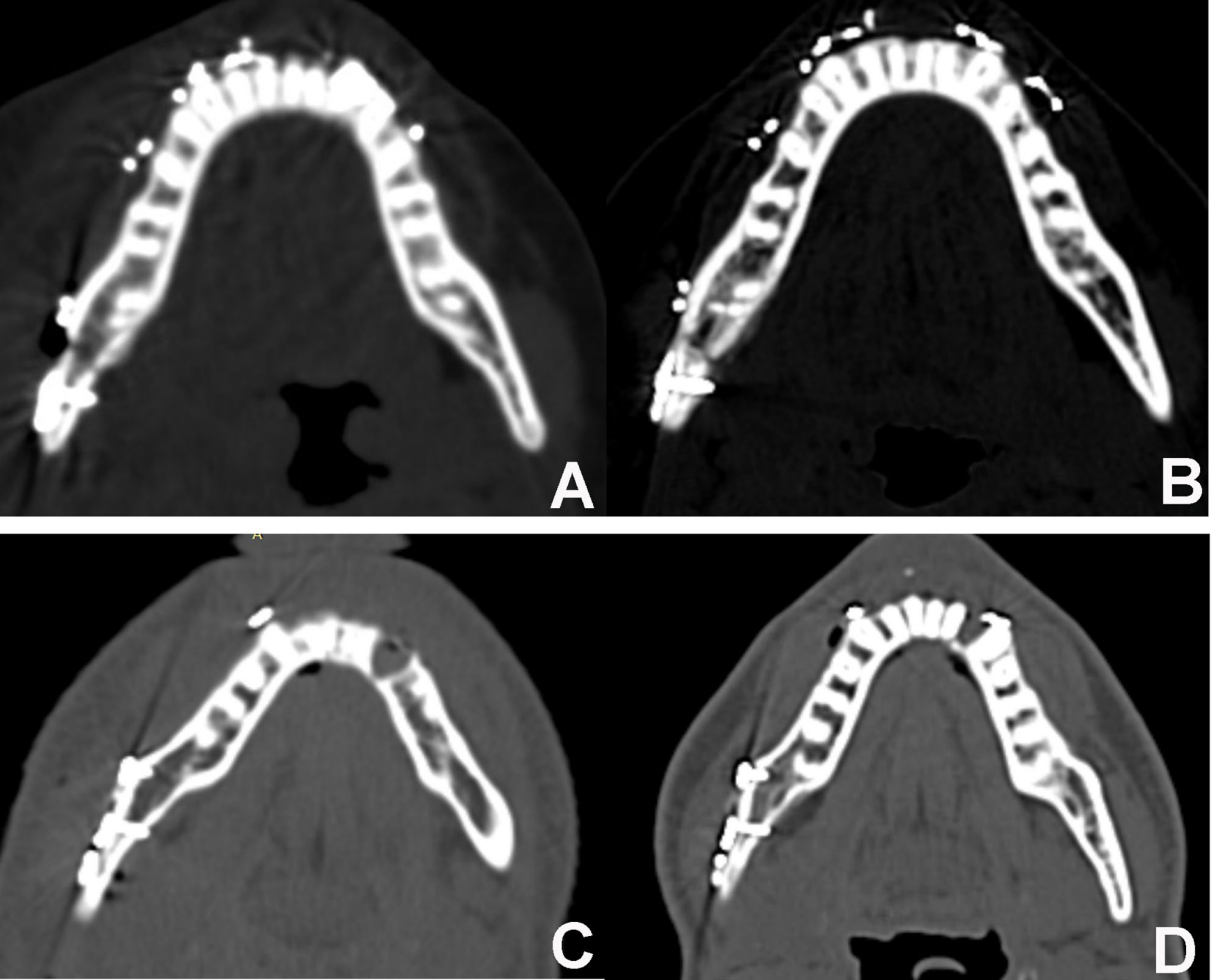
**A** Axial cut immediately postoperative of the study case **B** Axial cut 3 months postoperative of the study case. **C** Axial cut immediately postoperative of the control case. **D** Axial cut 3 months postoperative of the control case

Radiographic evaluation comprised immediate and 12-week postoperative computed tomography (CT) scans to assess reduction adequacy and quantify bone mineralization [28] (Fig. 6). To ensure the high reliability of the radiodensitometric analysis, all CT scans were acquired using the same Philips 16-detector CT scanner. Acquisition parameters were strictly standardized to mitigate inter-scan variability: tube voltage of 100–130 kVp (weight-adjusted), tube current of 150–175 mAs, and a slice thickness of 1 mm, with images reconstructed utilizing a high-resolution bone kernel. Furthermore, daily automated air and water calibrations were executed via the scanner’s internal quality assurance system, ensuring absolute stability and reproducibility of the Hounsfield Unit (HU) measurements across the study period. DICOM data were imported into MIMICS software to quantify bone healing in Hounsfield Units. To mitigate beam-hardening metal artifacts, three standardized Point-ROIs (superior, middle, and inferior thirds) were placed along the fracture line, strictly maintaining a 2-mm safety margin from all radiopaque hardware. The average of these three readings was calculated to determine the mean bone density for each scan (Fig. 3D). To ensure data reliability, measurements were performed by an independent blinded assessor. Inter-observer agreement was verified by a second radiologist on 20% of the sample, yielding an Intraclass Correlation Coefficient (ICC) > 0.88.

**Fig. 7:**
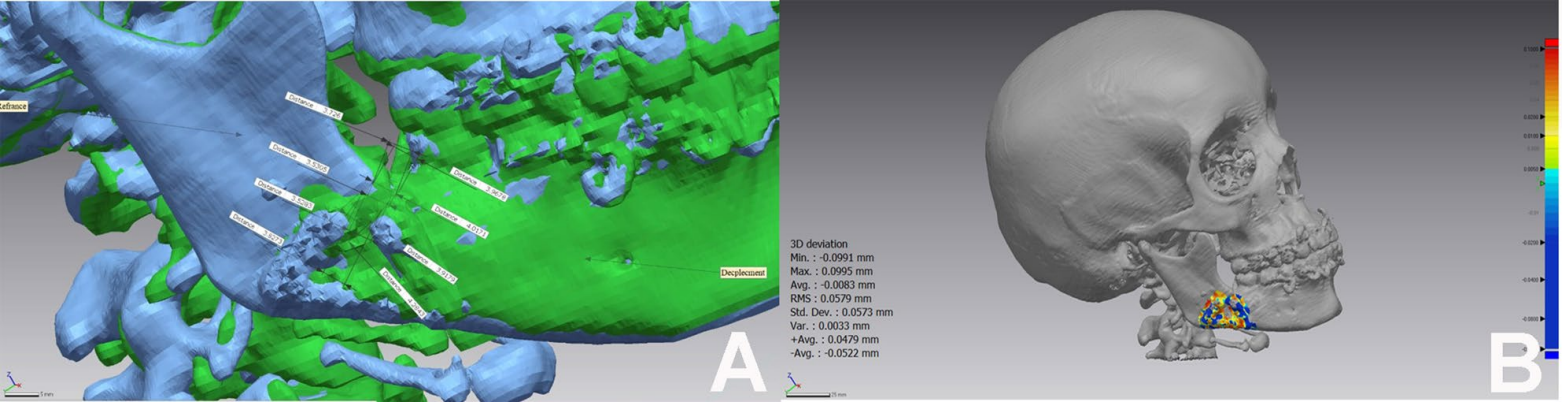
**A** 3D model of 3-months postoperative C.T displacement data (green) superimposed on the 3D immediate postoperative C.T Reference data (blue) to visually highlight the difference caused through follow up period under patient’s biting forces showing mean displacement of 0.3 mm. **B** Color mapping shows the root mean square (RMS) to determine the extent of SSOP deviation during the follow up period. The mean RMS value was 59 microns

To validate the mechanical integrity of the novel prototype in vivo, 3D superimposition was performed specifically for the Study Group. Using Geomagic Control X software, immediate and 3-month postoperative 3D models were superimposed to quantify any material deformation or hardware deviation under functional loading (Fig. 7A).

### Statistical analysis

All statistical analyses were performed using SPSS, version 23 for Windows (Armonk, NY, USA). Normality of continuous variables was checked using Shapiro Wilk test and they were summarized as mean ± standard deviation (SD) and median with interquartile range (IQR), while categorical variables were presented as frequencies and percentages. Baseline differences in age were assessed using an independent samples t-test, whereas categorical variables, including gender, mechanism of trauma, fracture laterality, associated fractures, and tooth involvement, were compared using Pearson’s Chi-Square or Fisher’s Exact test as appropriate. Postoperative pain, being non-normally distributed, was analyzed using the *Friedman test* for within-group changes over time, with *Wilcoxon Signed Rank tests* and Bonferroni correction applied for post-hoc pairwise comparisons, and *Mann–Whitney U tests* for between-group comparisons at each interval. Maximum mouth opening and bone density, measured repeatedly and normally distributed, were analyzed using *repeated measures ANOVA* to assess the main effects of Group, Time, and their interaction, with partial eta squared (η²) reported as a measure of effect size; Greenhouse–Geisser correction was applied when sphericity assumptions were violated. Dichotomous outcomes, including postoperative infection and malocclusion, were compared between groups using *Fisher’s Exact test*. Operative time was compared using an *independent samples t-test*. All tests were two-tailed, and *p*-values < 0.05 were considered statistically significant.

## Results

### Pre-clinical mechanical test results

Under vertical incisal loading (60 N), the hemimandible replicas fixated with the novel SSOP design exhibited a mean vertical displacement of 1.46 mm. Digital analysis of the pre- and post-loading 3D models revealed a Root Mean Square (RMS) deviation of 60 microns. These values indicated that the prototype possessed sufficient stiffness to resist significant deformation under loads simulating the early healing period (Fig. 3B, C).

### Clinical outcomes

**Fig. 8:**
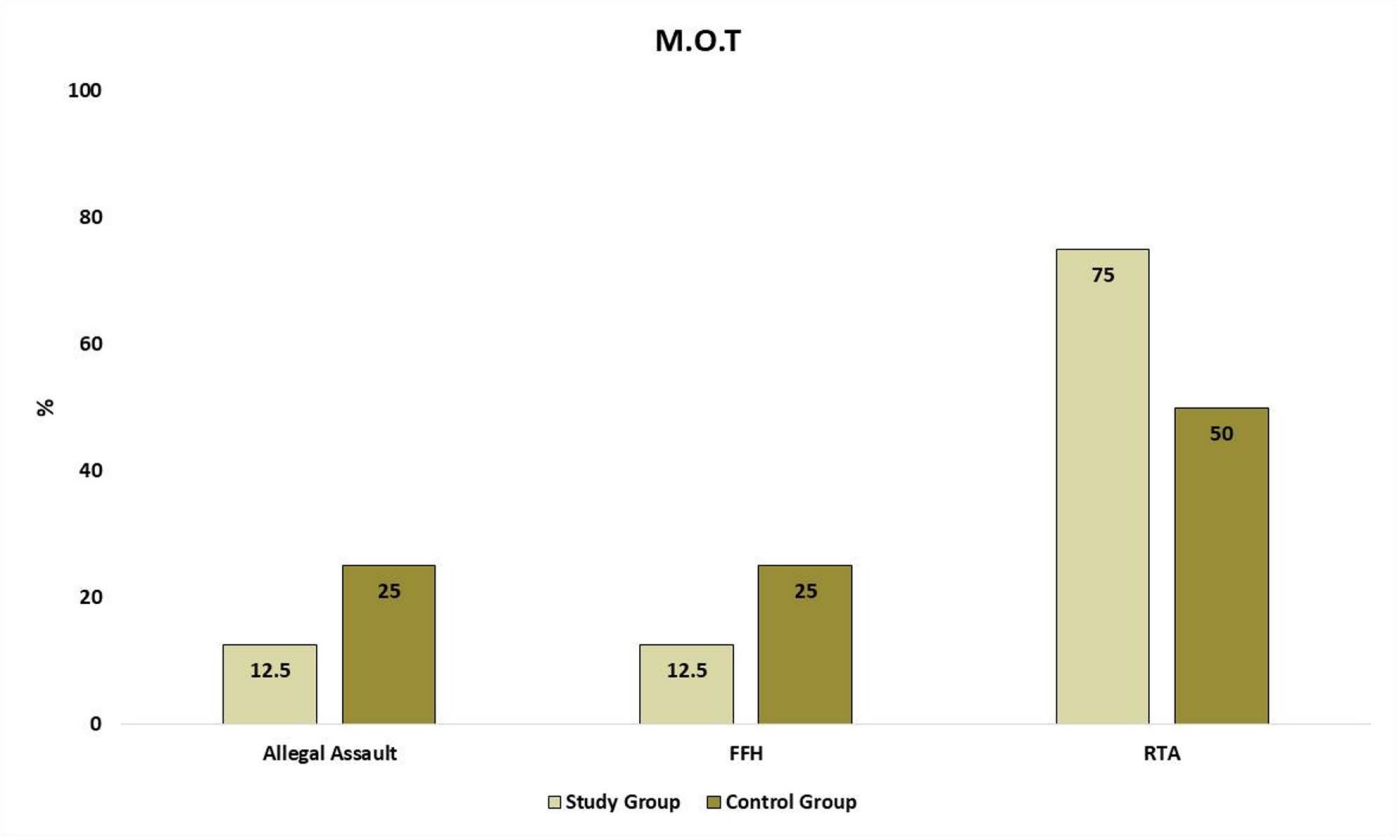
Comparison between study & control groups according to etiology of trauma

A total of 16 patients were randomized into the study group (novel SSOP, *n* = 8) and the control group (conventional two-miniplate fixation, *n* = 8). Demographic analysis revealed a male predominance in both groups; Study group comprised 7 males (87.5%) and 1 female (12.5%), whereas Control group included 6 males (75%) and 2 females (25%). The mean age was 31.50 ± 9.80 years in the study group and 30.50 ± 7.93 years in the control group. Regarding injury characteristics, angle fractures were evenly distributed between the right (*n* = 8) and left (*n* = 8) sides. Seven patients in each group presented with an associated contralateral fracture (parasymphysis or body). There was no significant intergroup difference in the distribution of these associated injuries. Furthermore, all contralateral fractures were treated with standard internal fixation using two miniplates prior to addressing the angle fracture to prevent biomechanical confounding. The etiology of trauma showed no significant variability between groups, comprising road traffic accidents (RTA) (62.5%), falls from height (18.75%), and alleged assaults (18.75%) (Fig. 8).

**Fig. 9:**
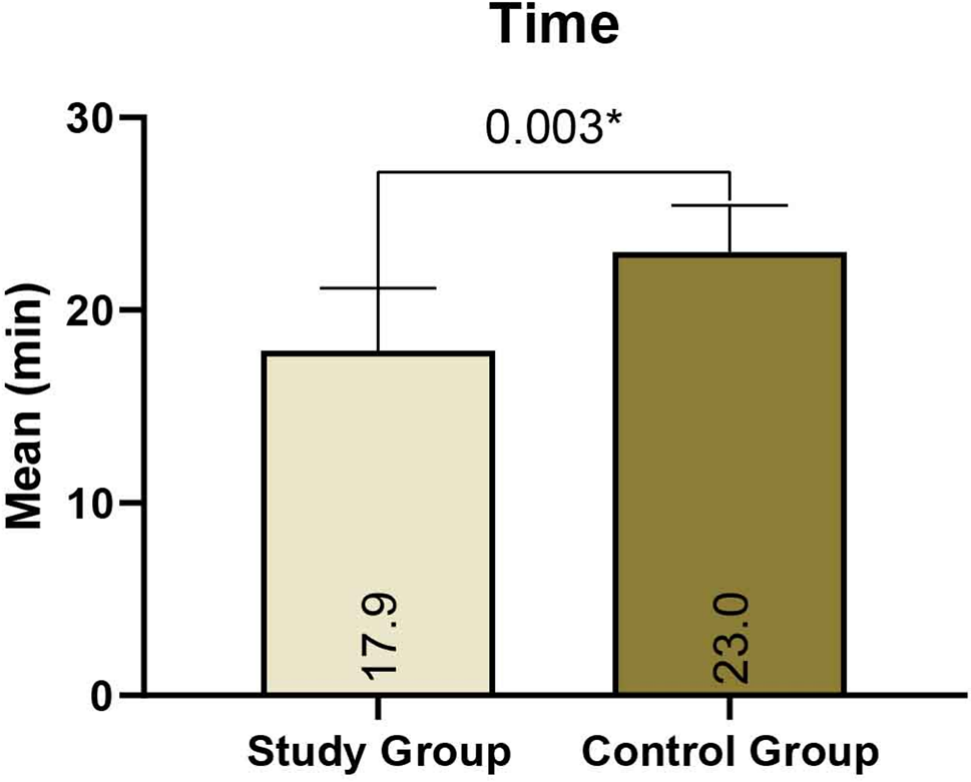
Comparison between the study & control groups according to operative time

Regarding postoperative functional recovery, occlusion was restored to pre-traumatic intercuspal relationships in all patients in the study group. One patient in the control group presented with mild early occlusal derangement during the first postoperative week for which intermaxillary fixation was done for one week and the occlusion corrected. At the 12-week follow-up, the mean maximal interincisal opening (MIO) was comparable between the study group (37.50 ± 6.89 mm) and the control group (37.00 ± 4.63 mm), with no statistically significant difference observed between the two groups. Both groups demonstrated a statistically significant progressive reduction in Visual Analogue Scale (VAS) pain scores over the 12-week follow-up period (*P* value < 0.001). Soft tissue healing progressed without complication across the study group. One case of wound dehiscence occurred in the control group during the first postoperative week; this localized infection resolved completely following conservative antibiotic therapy and local wound care. Regarding surgical efficiency, the mean operative time was significantly shorter for the study group (17.88 ± 3.27 min) compared to the control group (23.00 ± 2.45 min; *p* < 0.05) (Fig. 9).

**Fig. 10:**
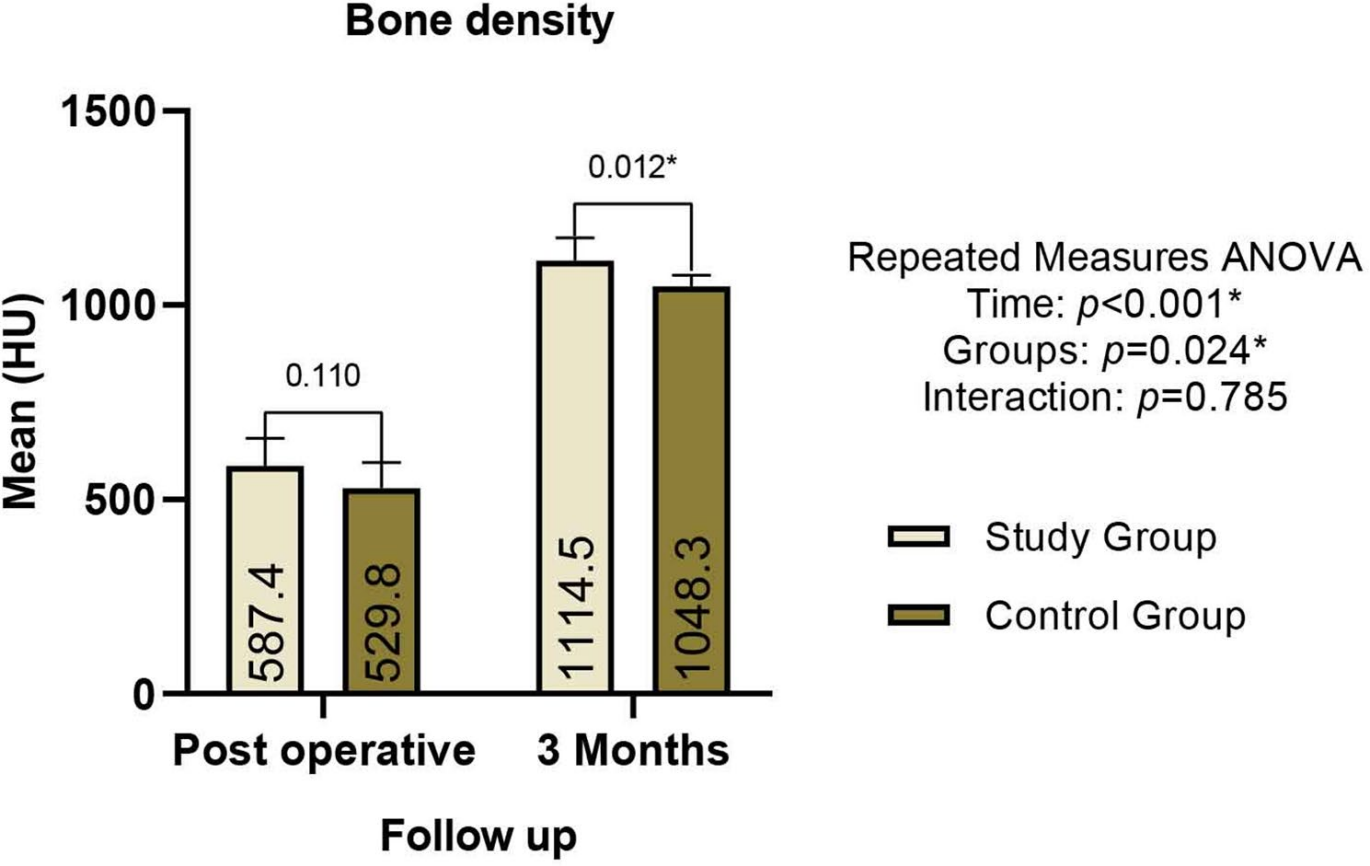
Comparison of mean bone density between the study and control groups during the follow-up period

Postoperative CT scans demonstrated anatomic fracture reduction with alignment of the mandibular borders. Follow-up imaging indicated osseous continuity, with no evidence of malunion or nonunion in any case. Longitudinally, both groups exhibited a progressive, statistically significant increase in intragroup bone density from the immediate postoperative phase to the 3-month follow-up (*p* < 0.001). Between-group comparisons revealed that while early density values were comparable, the study group recorded significantly higher mean bone density at the 3-month interval (*p* = 0.04) Table 1, (Fig. 10).

**Table 1:** Comparison of bone density between the study & control groups at different time intervals

	Study Group ***(n = 8)***		Control Group ***(n = 8)***		*p* value
	Mean ± SD	Median (IQR)	Mean ± SD	Median (IQR)	
Post Operative	587.41 ± 69.39	554.30 (106.17)	529.80 ± 65.73	518.30 (89.30)	0.110
3 Months	1114.46 ± 58.30	1128.65 (92.75)	1048.34 ± 28.86	1050.80 (46.50)	0.012^*^
*p* value	< 0.001^*^		< 0.001^*^		

*SD* Standard Deviation, *IQR* Inter Quartile Range

^*^Statistically significant difference at *p* value < 0.05

Regarding the in vivo validation of the novel SSOP prototype, 3D superimposition of the study group scans at 3 months revealed a mean root mean square (RMS) deviation of 59 μm and a mean hardware displacement of 0.3 mm. (Fig. 7B).

## Discussion

Mandibular angle fractures are anatomically challenging and historically prone to high complication rates [29–32]. To address this, our study evaluated the clinical and radiographic outcomes of a novel shape-optimized plate (SSOP) compared to conventional two-miniplate fixation, alongside pre-clinical biomechanical testing of the SSOP prototype.

This study observed a male predominance (> 80%), consistent with the 4.5:1 ratio reported by Melek et al. [33] and El-Mahallawy et al. [34]. The male predominance and high incidence of road traffic accidents observed in this study align with established demographic patterns for maxillofacial trauma in developing regions [35].

The novel SSOP design introduces a ‘Hybrid Fixation Concept’ that departs from strict adherence to Champy’s tension-band principle [36]. This design anchors the plate at the inferior border for bicortical screw purchase in dense basal bone, counteracting the distraction forces of the masticatory musculature [37], while avoiding the pull-out risks associated with monocortical screws [38, 39]. Vertical arms extend into the tension zone, creating a rigid “Four-Point Geometric Frame” to manage superior tensile and torsional forces by combining selectable hole placement with bicortical engagement achieving maximum 3D rigidity [40]. Unlike conventional linear miniplates susceptible to bucco-lingual splaying [37], this multi-point construct actively neutralizes multiplanar moments. To ensure a standardized baseline, the control group also utilized a dual-miniplate configuration at the inferior border.

Beyond pure biomechanics, the SSOP optimizes surgical adaptability. Manufactured from Grade 4 Titanium, the device provides a critical balance of rigidity and malleability. This allows for precise, passive intraoperative contouring to the external oblique ridge without “spring-back,” reducing the risk of iatrogenic injury during placement [41]. This material choice, combined with the plate’s broad-based geometric footprint, creates a “brace effect” that significantly enhances transverse stability (Z-axis) [39]. Furthermore, unlike 3D grids that require intimate bone contact across a broad surface [42], the SSOP functions as a “Bridging Construct.” This multi-point geometry grants the surgeon vital positional flexibility, permitting a “skip-hole” technique to safely bypass the inferior alveolar canal and impacted dental roots without compromising overall structural rigidity [43]. This adaptability allows for placement over complex or oblique fractures, offering a potential available option without the manufacturing delays or increased costs associated with complex 3D plates [44].

Radiographically, consistent with Melek et al. [33], both groups demonstrated progressive bone density increases, confirming that adequate stabilization was achieved in all cases, contrasting with the high complication rates (up to 28%) historically reported by Chrcanovic [45] using conventional non-compression miniplates. Crucially, this study rejected the null hypothesis regarding radiographic bone healing. The higher bone density observed in the study group at three months suggests that SSOP’s unique geometric frame may provide an optimized biomechanical environment for early osseous consolidation. While interpreted cautiously without histological confirmation, this superior mineralization suggests SSOP’s geometric stability successfully minimizes inter-fragmentary micromotion [46]. These findings align with Al-Moraissi et al. [42], who noted that geometric miniplates reduce postoperative complications compared to conventional linear designs. The use of novel shapes or alternative positioning configurations has the potential to offer even greater stability [47].

Clinically, while the study group achieved statistically superior radiographic bone density at three months, this was not mirrored by significant differences in pain or mouth opening. This discrepancy likely reflects a clinical “ceiling effect,” where the rapid resolution of acute symptoms masks subtle biomechanical advantages in healing quality [33]. The absence of a statistically significant difference in postoperative pain and maximal interincisal opening (MIO) between the cohorts is likely attributable to the standardized extraoral surgical approach employed in both groups [48, 49]. The progressive improvement in MIO aligns with similar longitudinal observations by Vineeth et al. [50] and Melek et al. [33].

Furthermore, patients with concomitant mandibular fractures were included and treated with standard internal fixation. Because the incidence of these secondary fractures was evenly distributed between the SSOP and control groups, their impact on clinical outcomes—such as pain and mouth opening—was balanced and did not confound the results. Regarding occlusal stability, the reproducible occlusion achieved in the study group while a single case in the Control group showed mild occlusal derangement, are consistent with the low malocclusion rates (0–8%) reported by Siddiqui et al. [47] and Ellis et al. [43]. However, Danda [51], reported comparable occlusal stability in studies comparing single versus two miniplates.

The new plate demonstrated statistically shorter operative time compared to the two-miniplate technique (*p* < 0.05). This finding concurs with Ellis et al. [37] and Jain et al. [52], who concluded that the application of a second plate significantly prolongs surgical duration. Furthermore, the single case of minor wound dehiscence in the dual-plate control group reflects broader literature trends; Essam et al. [42], Schierle et al. [53], and Sakkas et al. [54] consistently attribute the higher complication rates of double-plating techniques to the extensive periosteal stripping and compromised regional vascularity required for dual-site exposure. Moreover, as supported by Ellis et al. [40, 55], single-plate geometric fixation significantly reduces operative time, hardware costs, and surgical morbidity compared to traditional double-plating, while other studies confirm single plates are reliable with minimal complications (0–7.5%) [56].

An extraoral submandibular approach was employed to ensure uncompromised visualization of the inferior border and allow perpendicular instrumentation to the basal bone, maximizing screw-to-bone contact for the novel SSOP construct. While intraoral or transbuccal techniques are popular [17, 19, 57], the extraoral route provided necessary visualization for severely displaced fractures and minimized intraoral soft tissue trauma [58]. Our methodology emphasizes that surgical access must be dictated by fracture type and the need for anatomical precision rather than just complication rates.

Prior to clinical implementation, the structural reliability of the novel SSOP was validated through Polymeric Mandible Mechanical Testing (PMMT) to ensure the construct could withstand physiological loads without plastic deformation [59, 60]. To maximize clinical relevance, testing parameters were aligned with average postoperative incisor bite forces (~ 60 *N*) [61, 62]. Vertical incisal loading was specifically utilized to simulate the maximum bending moments experienced during the immediate postoperative soft-diet phase, during which the mandible acts as a Class III lever [63, 64]. While this configuration prioritizes the assessment of bending resistance over torsional deformation, it provides a clinically relevant assessment of the hardware’s stability during the most critical phase of secondary bone healing [65].

During incisal loading with average biting force = 60 *N*, the hemimandible replicas exhibited a mean displacement of 1.46 mm. (The observed displacement was a non-axial, multidirectional rotational motion with inferior and lingual components). These values are consistent with the findings of Ay et al. [65] and De Oliveira et al. [66] for miniplate fixation in mandibular angle fractures, confirming the SSOP’s capacity to safely neutralize functional loads prior to human trials.

Ultimately, while case-specific preoperative planning remains essential, this novel configuration offers a highly efficient, multiplanar solution that bridges the gap between conventional miniplates and complex 3D systems. However, each surgical modality has specific benefits and drawbacks, so case-specific approach driven by accurate diagnosis and preoperative planning is essential.

A primary strength of this investigation is its dual-phase methodology, integrating pre-clinical bench testing with in vivo digital tracing to empirically verify device stability. Notably, mechanical testing and 3D deviation analysis were conducted exclusively on the study cohort as a prototype validation tool rather than a comparative measure. Because a parallel control group was not included in the pre-clinical testing phase, no direct biomechanical superiority can be concluded. Consequently, intergroup efficacy was evaluated strictly through clinical and radiographic biological endpoints.

While the clinical stability observed in the Study group parallels the rigidity demonstrated during the pre-clinical validation phase, a direct statistical correlation could not be established due to the limited sample size of the in vivo model and the complexity of in vivo variables. However, these findings collectively suggest that the novel SSOP design possesses sufficient structural integrity to support bone healing under functional loads.

This preliminary study has several notable limitations. First, the small sample size of this single-center cohort, resulting from strict inclusion criteria, provided adequate power for the primary outcome of bone density but remains underpowered for clinical safety; thus, complication rates should be interpreted with caution (Type II error risk). Second, pre-clinical validation lacked a parallel control group and utilized isotropic resin models, which cannot perfectly replicate the anisotropic properties of human bone. Third, the 3-month follow-up captures initial consolidation but is insufficient to detect late-stage complications such as hardware failure or nonunion. Fourth, while including associated contralateral fractures accurately reflects typical trauma populations, it may introduce biomechanical confounding [67]. Finally, relying solely on CT Hounsfield Units without histological validation restricts the assessment to early mineralization; CT cannot differentiate between woven and mature lamellar bone, and residual beam-hardening artifacts may persist despite standardized 2-mm safety margins.

## Conclusion

Within the limitations of this study, the novel SSOP demonstrated reduced operative time and comparable short-term clinical outcomes to conventional double-miniplate fixation. Radiographically, higher bone density was observed at three months, and the prototype demonstrated acceptable baseline biomechanical outcomes during pre-clinical testing. While the SSOP represents a potential fixation alternative, larger longitudinal studies remain necessary to evaluate long-term clinical outcomes and to directly compare this prototype biomechanically against other established plates used for mandibular angle fractures.

## Supplementary Information

Supplementary Material 1.

## Data Availability

The datasets used and/or analyzed during the current study are available from the corresponding author on reasonable request.

## Abbreviations

SNOSE: Sequentially numbered, opaque, sealed envelopes
3D: Three-dimensional
ROI: Region of interest
CT: Computed Tomography
DICOM: Digital Imaging and Communications in Medicine
HU: Hounsfield Unit
IBM (SPSS): International Business Machines Statistical Package for Social Science
IMF: Inter Maxillary Fixation
MAFs: Mandibular angle fractures
MIMICS: Materialise mimics
Mm: Millimeters
μm: Microns
N: Newton
min: minutes
RIF: Rigid internal fixation
RTA: Road traffic accidents
SSOP: Sagittal split osteotomy plate
STL: StereoLithography
VAS: Visual Analogue Scale
CNC: Computer Numerical Control
